# Necrotizing Fasciitis Secondary to a Primary Suture for Anoperineal Trauma by Motorcycle Accident in a Healthy Adult

**DOI:** 10.1155/2015/956156

**Published:** 2015-08-10

**Authors:** Susumu Saigusa, Masaki Ohi, Hiroki Imaoka, Ryo Uratani, Minako Kobayashi, Yasuhiro Inoue

**Affiliations:** ^1^Department of Surgery, Wakaba Hospital, 28-13 Minami-Chuo, Tsu, Mie 514-0832, Japan; ^2^Department of Gastrointestinal and Pediatric Surgery, Mie University Graduate School of Medicine, 2-174 Edobashi, Tsu, Mie 514-8507, Japan

## Abstract

A 41-year-old man experienced a swollen scrotum three days after a motorcycle accident and presented to our hospital. He had had a primary suture repair for anoperineal trauma in an outside hospital at the time of the injury. He presented to us with general fatigue, low grade fevers, and perineal pain. Abdominal computed tomography showed subcutaneous emphysema from the scrotum to the left chest. The sutured wound had foul-smelling discharge and white exudate. We made the diagnosis of necrotizing fasciitis and immediately opened the sutured wound and performed initial debridement and lavage with copious irrigation. We continued antibiotics and lavage of the wound until the infection was controlled. Fortunately, the necrotizing fasciitis did not worsen and he was discharged after 15 days. Our experience indicates that anoperineal injuries should not be closed without careful and intensive follow-up due to the potential of developing necrotizing fasciitis.

## 1. Introduction

Necrotizing fasciitis (NF) is an uncommon, rapidly progressive soft tissue infection involving necrosis of subcutaneous tissues with an alarmingly high mortality rate. Appropriate diagnosis and prompt treatment are extremely important [1–4]. However, early recognition of NF is difficult clinically because there may only be minor skin changes such as local pain upon palpation and erythema in the early phases. Although there is no age or gender predilection, higher rates of NF are seen in obese, diabetic, and immunocompromised patients, as well as in alcoholics and patients with peripheral vascular disease. However, NF can also occur in young, otherwise healthy patients with none of these predisposing factors [1, 3, 5]. We report a case of necrotizing fasciitis after primary suture repair for an anoperineal injury by motorcycle accident with a favorable clinical outcome in a healthy adult.

## 2. Case Presentation

A 41-year-old Brazilian man with scrotal swelling presented to our hospital, thinking that perhaps he had a recurrence of an inguinal hernia which had been repaired at our hospital three years prior. Upon evaluation at our hospital, we discovered that at an outside hospital he had undergone primary suture repair of anoperineal trauma 3 days prior, secondary to a motorcycle accident. Cefalexin (300 mg/day) and a nonsteroidal anti-inflammatory drug were prescribed by the doctors at that initial presentation, and he was scheduled to return there five days after the repair for follow-up. At our hospital, he presented with general fatigue, low grade fevers, perineal pain, and crepitus from the left chest to the flank.

**Figure 1:**
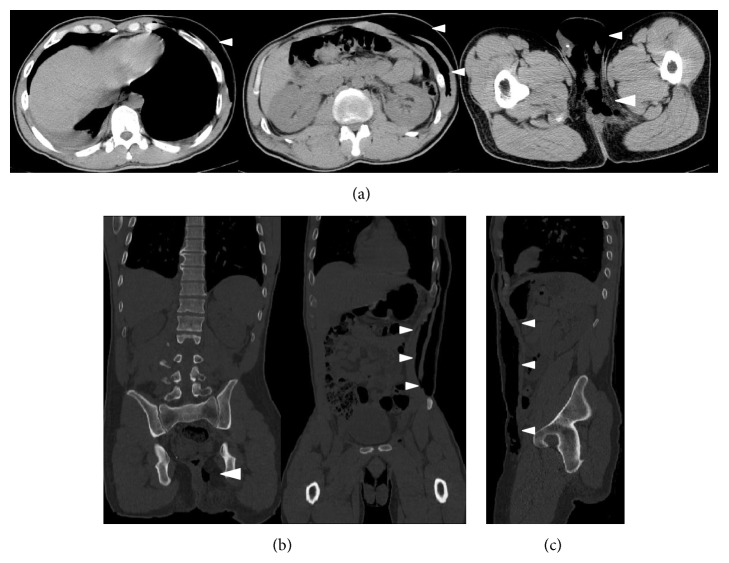
Abdominal CT on admission. Subcutaneous emphysema spreading from the scrotum to the left chest: plain CT (a), frontal section (b), sagittal section (c), and subcutaneous gas (arrow head).

**Figure 2:**
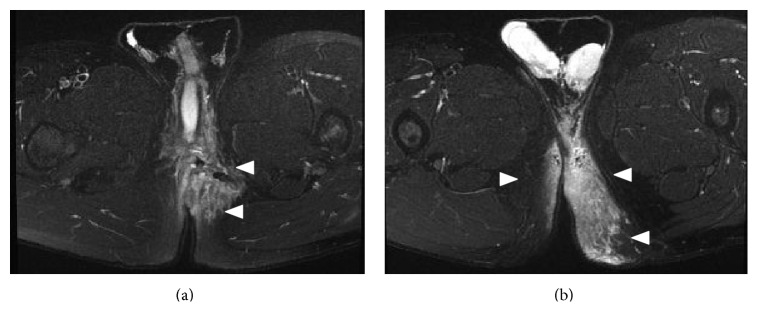
STIR sequence of the MRI showed high intensity around the perineum.

**Figure 3:**
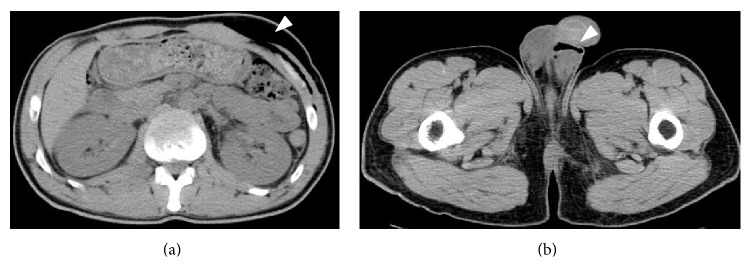
Abdominal CT after 10 days in the hospital. Subcutaneous gas remains in the left flank despite prompt disappearance of emphysema in the scrotum. Subcutaneous gas (arrow head).

Abdominal computed tomography (CT) showed that there was subcutaneous emphysema from the scrotum to the left chest (Figure 1), multiple fractures of bilateral ribs, a right pleural effusion, multiple transverse process fractures, fracture of the fourth lumber vertebral body, and a retroperitoneal hematoma; he had no recurrence of a right inguinal hernia. Neither pelvic fracture nor urethral injury was observed. Upon examination of the anoperineal area, the sutured and contused/lacerated wound had foul-smelling discharge and white exudate. Digital examination demonstrated reduced anal sphincter tone. Magnetic resonance imaging (MRI) showed high intensity around the perineum on short T1 inversion recovery (STIR) sequences, strongly suspicious for necrotizing fasciitis (Figure 2). Additionally, MRI suggested a left anal sphincter injury. We immediately opened the wound and performed an initial debridement and lavage with copious irrigation. On laboratory examination, abnormal values were as follows: white blood cell 11200/*μ*L (normal range, 3500–9000/*μ*L), platelet 11.8 × 10^4^/*μ*L (normal range, 14.0–37.9 × 10^4^/*μ*L), aspirate aminotransferase 43 IU/L (normal range, 10–35 IU/L), lactate dehydrogenase 265 IU/L (normal range, 110–225 IU/L), creatinine kinase 1054 IU/L (50–200 IU/L), and C-reactive protein 7.27 mg/dL (0–0.45 mg/dL). Wound cultures showed* Enterobacter cloacae*,* Pseudomonas aeruginosa*, and yeast. After admission to our hospital, we administered meropenem (2 g/day) and clindamycin (1800 mg/day), kept the patient NPO, and initiated parenteral nutrition. We continued antibiotics and wound lavage until the infection was controlled. Although low grade fever continued for ten days and subcutaneous gas remained in the left flank despite prompt disappearance of emphysema in the scrotum (Figure 3), the inflammation-related laboratory data improved gradually. He resumed oral intake after a week in the hospital. He required no further surgery and was discharged on hospital day 15. The subcutaneous air completely disappeared on abdominal CT two months after the first visit to our hospital. He had no fecal incontinence despite the injury to his sphincter. He is still followed carefully in the ambulatory setting.

## 3. Discussion

In the present case, early diagnosis and treatment prevented the progression of severe NF and led to a favorable clinical outcome. The patient visited our hospital 3 days after primary suture repair for anoperineal trauma. The typical symptoms and signs of NF are as follows: the wound develops tense edema extending beyond the margin of erythema with a wood-like feel, bullae, discoloration progressing to grey and necrotic skin, crepitus, and a broad erythematous tract in the skin along the route of the infection [1, 3, 5, 6]. We would not have suspected NF according to only macroscopic appearance of wound with foul-smelling discharge and white exudate because the appearances of NF in early phase are different. In our case, he did not understand why he had continued general fatigue and was also not aware of the subcutaneous gas with crepitus of his left chest and flank. If he had not suspected a recurrence of a prior inguinal hernia and visited our hospital promptly, his infection may have progressed into a life-threatening condition.

The risk factors for developing NF are skin injuries including insect bites, trauma, and surgical wounds, as well as underlying alcohol abuse, intravenous drug abuse, chronic liver or renal disease, diabetes, malignancies, and immunosuppression [1, 3, 4]. However, our patient had none of these predisposing factors. If primary suture for anoperineal trauma had not been done, he likely would not have suffered from NF because his clinical symptoms were immediately improved by initial debridement, wound irrigation, and open drainage. The laboratory risk indicator for necrotizing fasciitis (LRINEC) score is the most widely adopted scoring system [7]. Moreover, Wall et al. reported that WBC < 15000/*μ*L and a serum sodium level ≧135 mmol/L had negative predictive value of 99% and 90% sensitivity for detecting NSTIs [8]. However, these criteria could not identify NF in the present case. These criteria are insufficient tools for early diagnosis of NF. Therefore, a detailed history, establishment of the chronology of symptoms, and appropriate studies are important to diagnose early and developing NF.

On MRI, we observed high intensity around the perineum on the STIR sequence (a T2-weighted sequence). STIR reveals the presence of edema as an area with high intensity compared to the normal tissues [9, 10]. Arslan et al. reported that MRI is not reliable for the diagnosis of NF because while it has been shown to be fairly sensitive, it lacks specificity because tissue enhancement on T2-weighted imaging is frequently seen after trauma and other noninfectious inflammatory processes [11]. However, the evaluation of NF by STIR sequence of MRI may be a helpful tool for diagnosis after a detailed history and physical.

NF of the perineal, genital, or perianal regions is known as Fournier's gangrene (FG), which can spread to the abdominal wall, causing soft tissue necrosis and sepsis [12–14]. In these areas, wound care can be extremely difficult owing to wound contamination from stool. For this reason, patients with FG have often undergone diverting colostomy for infection control. Li et al. have reported that the use of enterostomy could significantly reduce the mortality rate in patients with FG [15]. In the present case, a diverting colostomy was not needed although we considered it upon initial presentation. However, the concurrent use of enterostomy for FG is still controversial [16, 17]. On the other hand, vacuum assisted closure (VAC) has been reported as a useful management for FG, resulting in reducing hospital stay and patient discomfort [18, 19]. In the present case, VAC therapy was not needed because the wound healing was prompt by conventional dressing.

In conclusion, our experience indicates that primary suture for anoperineal injury should not be performed without careful and intensive follow-up due to the potential for developing NF without any predisposing risk factors. Additionally, a less-experienced physician should consult with an experienced physician as to the treatments of anoperineal injury.
